# A Rare Case of Sarcomatoid Variant of Mixed Germ Cell Tumor of the Testis Presenting With Malignant Hypercalcemia and Tumor Lysis Syndrome

**DOI:** 10.7759/cureus.19749

**Published:** 2021-11-19

**Authors:** Sindhusha Veeraballi, Dilesha D Kumanayaka, Bader Omour, Amy Paige, Hamid Shaaban

**Affiliations:** 1 Internal Medicine, Saint Michael's Medical Center, Newark, USA; 2 Hematology/Oncology, Saint Michael's Medical Center, Newark, USA

**Keywords:** cancer, testicular germ cell tumors, tumor lysis syndrome, hypercalcemia, sarcomatoid

## Abstract

Hypercalcemia of malignancy is relatively common in several cancers. However, in testicular cancer, paraneoplastic hypercalcemia is uncommon. We describe the first case of severe tumor lysis syndrome associated with hypercalcemia from bone metastasis of testicular origin. Classically, tumor lysis syndrome is associated with hypocalcemia. This was a diagnostic and therapeutic challenge.

## Introduction

Hypercalcemia is a rare complication of malignant germ cell tumors [1]. The problem may be missed because of unawareness among internists and oncologists. It is even rarer to see significant hypercalcemia with normal parathyroid hormone and normal parathyroid hormone-related protein. The most common cancers associated with hypercalcemia are breast, renal, lung cancer and multiple myeloma. Malignancy that coincides with hypercalcemia on initial presentation is associated with poor prognosis [2,3]. Testicular carcinoma is rarely associated with malignant hypercalcemia and tumor lysis syndrome, due to early diagnosis leading to good prognosis in most cases [2-8]. Herein, we present a unique case of malignant hypercalcemia secondary to metastatic sarcomatoid variant of mixed germ cell testicular carcinoma in a young male, who did not seek timely medical attention and presented at an advanced stage.

## Case presentation

A 25-year-old male with no significant past medical history presented with abdominal pain, significant nausea and vomiting for two to three months but progressively worsening for five days prior to admission. Pain was described as sharp with radiation to the back, associated with significant anorexia, constipation, dyspnea and intermittent chest pain. Additionally, the patient admitted to bilateral testicular enlargement, anorexia and significant weight loss of approximately 30 pounds over the prior two years.

On admission, vital signs were unremarkable except for a temperature of 101.8F. On physical exam, the patient appeared cachectic, in moderate distress secondary to diffuse abdominal and back pain. There was bilateral testicular enlargement (~15cm) with no erythema but with mild tenderness and no gross lymphadenopathy. A large right-sided abdominal mass was palpated. Labs (Table 1) were significant for elevated creatinine consistent with an acute kidney injury at 2 .0 mg/dL. Phosphorus was elevated at 4.9 mg/dL but potassium was within normal limits. He had severe hypercalcemia with a calcium level significantly elevated at 14.4 mg/dL with a low parathyroid hormone of 2.7 pg/mL. Lactic acid was 1.9 mg/dL. Uric acid was considerably elevated at 15.8 mg/dL. Alpha fetoprotein was significantly elevated at 716 ng/mL, lactate dehydrogenase was increased at 772 U/L with normal human chorionic gonadotropin (HCG) <0.1 mIU/mL.

**Table 1:** Admission laboratory results CBC: complete blood count, CMP: comprehensive metabolic panel

CBC	Result	Reference Interval
White Blood Count (10^3^/uL)	13.40	4.40 - 11.00
Red Blood Cell(10^6^/uL)	4.83	4.32 - 5.72
Hemoglobin (g/dL)	12.2	13.5 - 17.5
Hematocrit (%)	37.1	38.8 - 50.0
Mean Corpuscular volume (fL)	76.8	81.2 - 95.1
Mean Corpuscular Hemoglobin (pg)	25.2	27.5 - 33.2
Mean Corpuscular Hemoglobin Concentration (g/dL)	32.8	33.4 - 35.5
Red Cell Distribution Width (%)	14.7	11.8 - 15.6
Platelets (10^3^/uL)	149	150 - 450
Mean Platelet Volume (fL)	10.7	7.4 - 11.0

CMP	Results	Reference Interval
Sodium (mmol/L)	133	136 - 145
Potassium (mmol/L)	3.8	3.5 - 5.3
Chloride (mmol/L)	93	98 - 110
Carbon dioxide (mmol/L)	30.0	20.0 - 31.0
Blood Urea Nitrogen (mg/dL)	33.0	6.0 - 24.0
Creatinine (mg/dL)	2.0	0.6 - 1.2
Glomerular Filtration Rate (mL/min/1.73m^2^)	50.0	>60
Blood Urea Nitrogen/Creatinine	16.5	20:1
Glucose (mg/dL)	88	70 - 140
Calcium (mg/dL)	14.4	8.6 - 10.4
Aspartate Transaminase (U/L)	36	10 - 36
Alkaline Transaminase (U/L)	23	9 - 46
Alkaline Phosphatase (U/L)	102	40 - 115
Total Protein (g/dL)	8.5	6.4 - 8.4
Total Bilirubin (mg/dL)	0.6	0.2 - 1.2
Albumin (g/dL)	3.9	3.6 - 5.1
Anion Gap (mmol/L)	10.0	5 - 15

Tumor Markers	Results	Reference Interval
Lactate Dehydrogenase (U/L)	772	122 - 222
Alpha-Fetoprotein (ng/mL)	716.2	0 - 2.5
Human Chorionic Gonadotropin (mlU/mL)	N	0 - 3

Test for Tumor Lysis Syndrome	Results	Reference Interval
Potassium (mmol/L)	3.8	3.5 - 5.3
Phosphorus (mg/dL)	4.9	2.1 - 4.3
Calcium (mg/dL)	>15.0	8.6 - 10.4
Uric Acid (mg/dL)	15	4.0 - 8.0

Chest X-Ray revealed a well marginated 1.5 cm nodule projecting over the left lower chest. Scrotal ultrasound showed an 11.4 cm heterogeneous mass in the right scrotum, which was highly suspicious for malignancy. A CT scan of the Chest/Abdomen/Pelvis showed a large right-sided retroperitoneal mass measuring up to 16 cm, with areas of suspected necrosis/hemorrhage with mass effect on surrounding structures causing moderate to severe right hydronephrosis; multiple lung nodules and multiple lytic lesions within the sternum, spine, and pelvis were also noted. Bone scan showed diffuse osseous metastatic disease.

**Figure 1:**
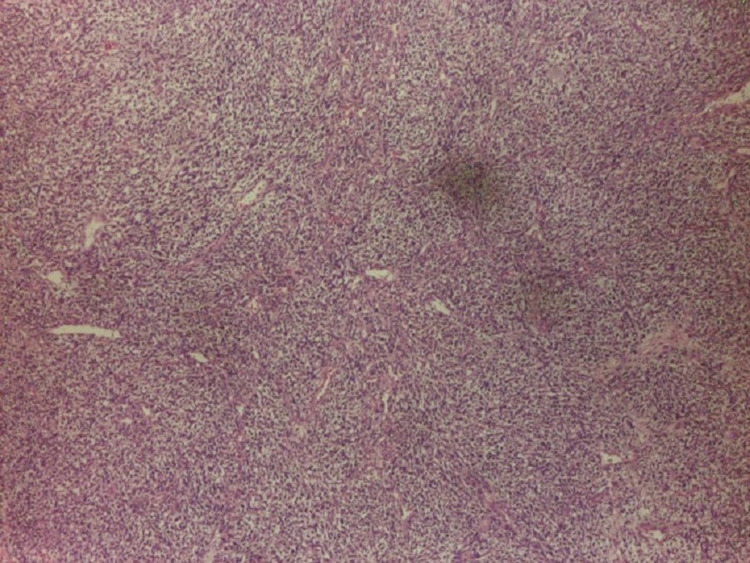
Sarcomatoid component of mixed germ cell tumor of testes (H&E stained)

**Figure 2:**
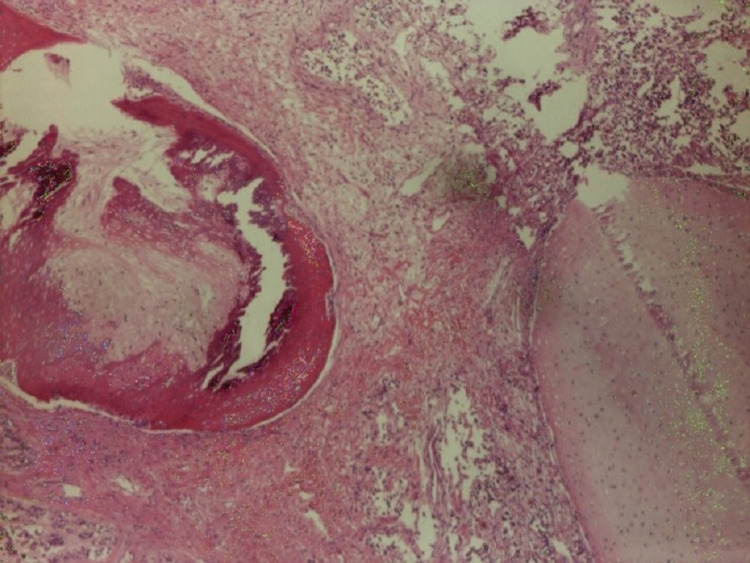
Teratoma component of mixed germ cell tumor of testes (H&E stained)

**Figure 3:**
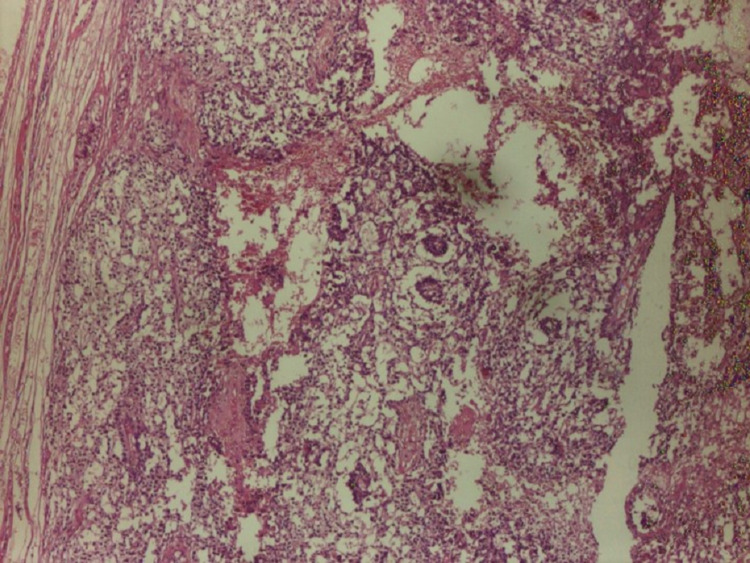
Yolk sac component of testicular cancer (H&E stained)

Aggressive intravenous fluids, pamidronate and calcitonin were immediately administered for hypercalcemia. He received allopurinol in view of spontaneous tumor lysis and high risk of progression of tumor lysis syndrome due to extensive tumor burden. Patient underwent total right radical orchiectomy along with right ureteral stent placement. Pathology was consistent with mixed germ cell tumor (80% yolk sac and 20% teratoma) with extensive sarcomatoid tumor transformation (rhabdomyosarcoma) (Figures1, 2, 3).

Creatinine and uric acid levels normalized and hypercalcemia was significantly improved over the next several days. Patient was subsequently discharged home. He was re-admitted again to intensive care unit for the management of hypercalcemia of >15 mg/dL. This time his labs demonstrated severe anemia with hemoglobin 6.6 mIU/mL and platelet count of 42 thousand platelets per microliter. Patient was transfused with 3 units of packed red blood cells and 1 unit of platelets. He also received pamidronate, aggressive hydration and calcitonin. He received his first cycle of chemotherapy with ifosfamide, cisplatin and etoposide.

## Discussion

Testicular cancer is the most common solid organ malignancy of men in the age group of 15-30 years [1-4]. The cause of germ cell testicular cancer is not completely understood [6,7]. A number of inherited defects have been reported to pose increased risk for developing germ cell testicular cancers including the central nervous system and genitourinary tract malformations and major malformations of the lower spine [8-12]. Specifically, males with cryptorchidism failure of the testes to descend into the scrotal sac), impaired spermatogenesis, inguinal hernia, hydrocele, disorders of sex development, prior testicular biopsy, atopy and testicular atrophy [13,14]. It has also been proposed that low testosterone levels, which result from somatic cells malfunctioning in the developing testes can also lead to germ cell testicular malignancies. The possible role of endocrine disrupting chemicals as risk factors has also been postulated [15]. Our patient did not report having any of the above mentioned predisposing factors.

Based on the data from SEER 18 2011-2017, the estimated five-year survival rate of testicular cancer is 94.9% [3]. This excellent prognosis is due to the combination of surgical resection and sensitivity of germ cell tumors to platinum based chemotherapy. The two main types of testicular cancer include germ cell tumors, which account for the 90% of testicular tumors, and sex cord stromal tumors. Based on histology, germ cell tumors are classified into seminomatous (SGTs) and non seminomatous germ cell tumors (NSGTs). NSGTs are further classified into yolk sac tumors, teratoma, trophoblastic tumors and embryonal tumors [4-6]. Thirty-two percent to 60% of NSGTs present as mixed germ cell tumors. About 60% of NSGTs present as advanced stage disease which can be attributed to insidious development, lack of overt signs, lymphatic and hematogenous metastasis of NSGTs [7].

Ours is a case of mixed germ cell tumor with co-occurrence of malignant hypercalcemia and tumor lysis syndrome as initial presentation, due to delay in seeking medical attention. Germ cell tumors are rarely associated with malignant hypercalcemia. Malignant hypercalcemia (MH) and tumor lysis syndrome (TLS) are two major life-threatening oncologic emergencies. MH is seen in 20-30% of patients with malignancy and most commonly seen in multiple myeloma, breast, lung cancer and hematologic malignancies like non-Hodgkin lymphoma, leukemia [7,8]. Multiple theories were proposed to explain the mechanism of hypercalcemia in malignancy which includes parathyroid hormone (PTH)-related peptide (PTHrP) mediated, osteolysis secondary to bone metastasis, ectopic PTH secretion, 1,25 dihydroxy vitamin D secretion [7-9]. Osteolytic metastases possibly explain malignant hypercalcemia in our case given the normal PTHrP and calcitriol levels with low PTH levels. The mainstay of treatment of malignant hypercalcemia includes intravascular volume repletion followed by diuresis, inhibiting bone resorption and enhancing renal calcium excretion. The definitive treatment is always the treatment of underlying malignancy [9]. Our patient received intravenous hydration, bisphosphonates and calcitonin as described above in the case summary to which the patient responded.

Tumor lysis syndrome occurs either spontaneously or after chemotherapy. It is commonly seen in rapidly proliferating hematological malignancies and is sometimes seen in germ cell tumors (GCTs). However, spontaneous onset of TLS in solid tumors is very rare. In a retrospective and pooled study conducted on TLS in GCTs, 17 cases with median age of 34 were identified in which eight cases (47%) had spontaneous tumor lysis. The higher incidence of STLS in GCTs can be attributed to the highly proliferative nature of these tumors. The mortality rate was 44.4% and 37.5% among cases of treatment-related TLS and STLS respectively [5].

There is a need to bring awareness regarding the risk of STLs for proper monitoring of patients and timely management of this life-threatening complication. Treatment of TLS is mainly prevention based on risk stratification [10] and initiating timely therapy with aggressive hydration measures, correcting electrolyte and metabolic disturbances, and maintaining adequate renal perfusion. Initiating allopurinol or rasburicase is case-dependent based on the index of suspicion and in tumors with high risk for spontaneous or chemotherapy-induced TLS. Our patient received allopurinol starting on day 1 given his tumor burden and responded to the measures described above. Studies have shown that surgical resection and platinum-based chemotherapy for germ cell tumors carries excellent prognosis with overall five-year survival of 94.9% [2]. In our case, the patient did not seek medical attention until the malignancy was at an advanced stage and already metastasized. He received one cycle of BEP (bleomycin, etoposide and cisplatin) and another cycle of palliative chemotherapy with VIP (etoposide, ifosfamide and cisplatin). Patient’s prognosis remains guarded at this time.

In TLS, intracellular calcium binds with phosphorus leading to hypocalcemia [6]. However, in our case persistent hypercalcemia was found associated with spontaneous TLS which made our case unique. Though hypocalcemia is usually seen with TLS, in tumors with high turnover, metastatic burden, or sensitivity to chemotherapy, hypercalcemia can be rarely seen [9-12] like in our case. We strongly suggest that the presence of hypercalcemia should not delay the diagnosis and initiation of treatment of TLS. Studies are required to carefully explore the pathological basis of malignant hypercalcemia in advanced germ cell tumors that have metastasized. More studies are also required to explore the etiology of TLS, whether it is initiated in utero and whether lifestyle choices, such as diet and physical activity, can decrease the risk of developing the tumor and whether environmental components play a role as predisposing factors [14,15].

## Conclusions

To our knowledge, this is the first reported case of tumor lysis syndrome and concomitant severe hypercalcemia in a patient with advanced sarcomatoid variant of mixed germ cell tumor. This unique case highlights the importance of seeking medical attention and initiating treatment early in the course of non-seminomatous germ cell tumors to improve prognosis. We strongly suggest that the presence of hypercalcemia should not delay the diagnosis and initiation of treatment of tumor lysis syndrome. Aggressive management with intravenous hydration and allopurinol must be prioritized given the poor prognosis associated with severe malignant hypercalcemia in this clinical setting. It is ideal to correct all the metabolic abnormalities before starting the anticancer treatment to achieve a better clinical outcome.
